# Investigation of Oncogenic Gene Mutation and Associated Protein Expression in Ameloblastic Carcinoma

**DOI:** 10.1111/odi.70420

**Published:** 2026-07-15

**Authors:** Puangwan Lapthanasupkul, Jintana Pankam, Siribang‐on Piboonniyom Khovidhunkit, Prapapun Seneetuntigul, Nakarin Kitkumthorn

**Affiliations:** ^1^ Department of Oral and Maxillofacial Pathology, Faculty of Dentistry Mahidol University Bangkok Thailand; ^2^ Oral Biology and Integrative Biomedical Science Program, Faculty of Dentistry Mahidol University Bangkok Thailand; ^3^ Department of Advanced General Dentistry, Faculty of Dentistry Mahidol University Bangkok Thailand; ^4^ Institute of Pathology, Department of Medical Services Ministry of Public Health Bangkok Thailand; ^5^ Department of Oral Biology, Faculty of Dentistry Mahidol University Bangkok Thailand; ^6^ Faculty of Dentistry Burapha University Chon Buri Thailand

**Keywords:** ameloblastic carcinoma, immunohistochemistry, MAPK/ERK pathway, oncogenic gene mutation, PI3K/mTOR pathway

## Abstract

**Objective:**

This study aimed to investigate gene mutations in ameloblastic carcinoma (AC) including *BRAF*, *KRAS*, and *PIK3CA*, along with associated protein expressions of BRAF V600E, p‐ERK1/2, and p‐mTOR, which represent activation of MAPK/ERK and PI3K/mTOR pathways, respectively.

**Materials and Methods:**

Ten formalin‐fixed, paraffin‐embedded AC tissue samples were collected. Tumor area samples were manually micro‐dissected for DNA extraction. All samples then underwent gene amplification using polymerase chain reaction and were subsequently sent for DNA sequencing. The investigation of protein expressions was assessed using immunohistochemistry.

**Results:**

Heterozygous mutations in *BRAF*, *KRAS*, and *PIK3CA* were observed in 40.0%, 30.0%, and 30.0% of AC cases, respectively. Notably, 75.0% of *BRAF*‐mutated cases (3/4) co‐harbored either *KRAS* or *PIK3CA* mutations. Immunohistochemical analysis revealed BRAF V600E expression in 40.0% of cases. AC also expressed mild immunostaining for p‐ERK1/2 (mean IRS ± SD; 2.50 ± 2.526) and moderate immunostaining for p‐mTOR (mean IRS ± SD; 7.62 ± 2.869), within both the cytoplasm and nucleus of the tumor cells.

**Conclusion:**

The presence of these oncogenic gene mutations and protein expressions suggests that MAPK/ERK and PI3K/mTOR pathways may contribute to AC pathogenesis. However, further studies with larger sample sizes are needed to confirm these findings.

## 1 Introduction

Ameloblastic carcinoma (AC) is a rare and highly aggressive malignant tumor of epithelial odontogenic origin, making it the most common type of odontogenic malignancy, accounting for about 30% of cases (World Health Organization 2022). It can develop spontaneously or evolve from a pre‐existing ameloblastoma (AM) or odontogenic cyst (World Health Organization 2022). AC affects a broad age range, with a median age of 49 years, and is more frequently observed in males. It is typically found in the posterior mandible, often presenting as an ill‐defined, expanding lesion associated with bone destruction (World Health Organization 2022). Notably, the rate of tumor recurrence increases, and the survival rate decreases with prolonged follow‐up (Robinson et al. 2024). The recurrence of AC has been strongly associated with conservative primary treatments, highlighting the need to avoid such procedures in the management of this aggressive cancer (Chrcanovic et al. 2022). The study of tumorigenesis at the gene and protein expression remains variable, particularly in rare cancers such as AC. Molecular alterations, including dysregulation of the Wnt/β‐catenin pathway, epithelial‐to‐mesenchymal transition‐related factors, and stemness‐associated markers such as SOX2, have been reported to contribute to the aggressive phenotype of AC (Cristofaro et al. 2025). However, the mechanisms underlying malignant tumorigenesis of AC remain poorly understood and warrant further investigation. Elucidating these molecular pathways could facilitate the development of targeted therapies. In contrast to other cancers, where mutations in key pathways have led to effective personalized treatments, AC currently lacks such therapeutic options. An understanding of its oncogenic pathways is therefore essential, not only to improve diagnostic accuracy but also to guide the development of more effective treatment strategies.

Previously, alterations in oncogenic signaling pathways, such as MAPK/ERK, Beta‐Catenin, PI3K, JAK/STAT, and Hedgehog (Hh) pathways, were commonly studied as they played a critical role in the development and progression of tumors and malignancies (Sanchez‐Vega et al. 2018). It is well established how these oncogenic signaling pathways exert their effects and how key mutations within them drive cancer development (Sanchez‐Vega et al. 2018).

The MAPK/ERK signaling pathway is a well‐established cascade involved in the regulation of cell proliferation, differentiation, and survival. Key components of this pathway, including RAS, RAF, ERK, and MAP kinases, are commonly investigated in odontogenic tumors (Oh et al. 2022). Previous studies have reported that the *BRAF V600E* mutation was frequently observed in AM (Togni et al. 2022; Chen et al. 2024; Tun et al. 2025). Furthermore, odontogenic sarcoma, a rare mixed tumor histologically characterized by benign ameloblastic epithelium within a malignant mesenchymal stroma, has also been found to harbor *NRAS* and *BRAF V600E* mutations (Buettner and Gultekin 2022). However, the role of the *BRAF V600E* mutation as a definitive diagnostic marker for AC has not yet been conclusively established (Soluk‐Tekkesin and Wright 2022). Interestingly, the *KRAS A146V* mutation was also found in 2 of 5 AC cases (Togni et al. 2022). Therefore, further studies on the activation of the MAPK/ERK signaling pathway are needed to explore which molecules play a crucial role in AC pathogenesis.

The PI3K/mTOR signaling pathway is one of the key regulatory pathways involved in cell proliferation and apoptosis. Genetic alterations and biochemical activation of this pathway have been frequently observed in both early and advanced stages of various cancers (Li et al. 2012). With regard to odontogenic tumors, overexpression of proteins associated with the PI3K/mTOR pathway, such as AKT and PI3K, has been suggested to contribute to the oncogenesis of odontogenic epithelium, including in AM (Kumamoto and Ooya 2007). Moreover, nuclear p‐mTOR expression has been reported to be higher in recurrent AM cases compared to those with cytoplasmic p‐mTOR expression, indicating a possible association with tumor aggressiveness or recurrence (Li et al. 2012). Although direct evidence from studies on AC remains limited, given the histopathological and molecular similarities between AM and AC, we hypothesized that this pathway might also be relevant in AC.

This study aims to investigate gene mutations such as *BRAF*, *KRAS*, and *PIK3CA*, as well as associated protein expressions including BRAF V600E, p‐ERK1/2, and p‐mTOR that are associated with the MAPK/ERK and PI3K/mTOR signaling pathways in AC. The study aims to provide new insights into the tumorigenesis of AC and to enhance our understanding of this rare and aggressive malignancy.

## 2 Materials and Methods

### 2.1 Samples Recruitment

This study was approved by the Faculty of Dentistry and Faculty of Pharmacy, Mahidol University, Institutional Review Board (COA no. MU‐DT/PY‐IRB 2023/071.1411). All procedures performed in this study were conducted in accordance with the Declaration of Helsinki. A total of 12 formalin‐fixed, paraffin‐embedded (FFPE) tissue blocks of AC, along with corresponding clinical data, were retrieved from the Department of Oral and Maxillofacial Pathology, Faculty of Dentistry, Mahidol University, Thailand, covering cases diagnosed between 2007 and 2022. Selected samples were reviewed to confirm the diagnosis of AC by a board‐certified oral pathologist (PL) according to the WHO classification (World Health Organization 2022) and had to contain at least 80% tumor cells. Samples with insufficient tumor area or suboptimal DNA quality were excluded from the study. Finally, only 10 cases were included in this study.

Regarding the histopathological diagnosis (Figure 1), AC shares similarities with AM but exhibits variable malignant features. Its architecture can range from follicular or plexiform patterns to solid basaloid sheets or anastomosing strands with loss of stellate reticulum. Diagnostic cytological features often include moderate to marked cellular or nuclear atypia, increased mitotic activity, high nuclear‐to‐cytoplasmic ratio, hyperchromatic vesicular nuclei, and basal cell crowding with disordered polarity. Central necrosis in islands is a valuable diagnostic clue (World Health Organization 2022).

**FIGURE 1:**
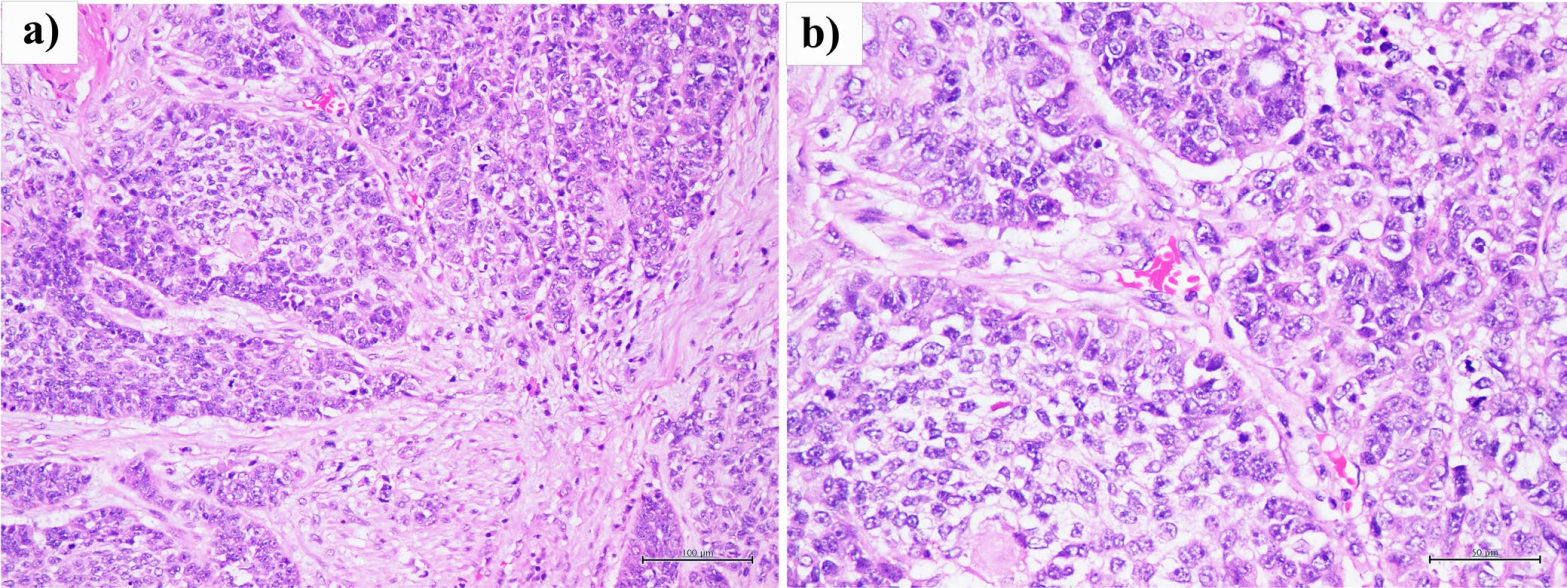
Histopathological feature of AC; hematoxylin and eosin staining (H&E) at 200× (a) and 400× (b) magnification.

### 2.2 Microdissection and DNA Extraction

Five‐micron sections of AC tissue were prepared, with about 10 slices per sample for microdissection (Kitkumthorn and Mutirangura 2010). Hematoxylin and eosin (H&E) slides were used as a reference to label the tumor areas. The tumor regions on the section slides were outlined with a permanent marker. After mapping, the tumor regions were manually micro‐dissected by carefully scraping the marked tumor areas from the section slides using a sterile 0.5 cm stainless steel spatula. The same spatula was then used to collect the dissected tissue fragments into microcentrifuge tubes for subsequent DNA extraction.

DNA isolation was performed using the standard phenol‐chloroform method (Sambrook and Russell 2001). The DNA pellet was resuspended in distilled water. DNA quality was assessed using a Nanodrop machine (Thermo Scientific, ND2000USCAN, USA). DNA samples with a concentration of around 100 ng/μL and an optical density ratio (OD) of approximately 1.8 at 260/280 nm were selected for gene amplification.

### 2.3 Gene Amplification and DNA Sequencing Analysis

The amplification of *GAPDH* was performed as a quality control measure for DNA samples. The PCR reaction mix consisted of a master mix containing Taq DNA polymerase (Thermo Scientific, Vilnius, Lithuania) and 100 ng/μL template DNA. We identified commonly reported mutations in cancer within *BRAF* exon 15 (Davies et al. 2002), *KRAS* exon 2 (Vogelstein et al. 1988), and *PIK3CA* exon 9 (Samuels et al. 2004). The primer sequences and corresponding conditions are detailed in Table 1, and the amplification process was carried out for 40 PCR cycles. Normal FFPE fibrous tissue was used as the positive control, while sterile distilled water was used as the negative control. Each PCR product was analyzed using 1% agarose gel electrophoresis stained with Intron Redsafe and visualized under UV transillumination using a Geldoc system (Bio‐Rad, 165 CA, USA).

**TABLE 1:** The primer sequences and conditions for amplifying target DNA.

Primers	Sequences (5′–3′)	Annealing temperature (°C)	DNA size (bp)
GAPDH	Forward: TGAGGCTCCCACCTTTCTCATC Reverse: TGAGGCCCTGCAGCGTACTC	56	157
BRAF	Exon 15 Forward: GAAATTAGATCTCTTACCTAAACTCTTCATA Reverse: GACCCACTCCATCGAGATTT	52	168
KRAS	Exon 2 Forward: AAAGGTACTGGTGGAGTATTTGA Reverse: TCAAGGCACTCTTGCCTACG	58	157
PIK3CA	Exon 9 Forward: CCAGAGGGGAAAAATATGACA Reverse: CATTTTAGCACTTACCTGTGAC	58	196

Positive PCR products were sent to Macrogen Inc. (Geumcheongu, Seoul, Republic of Korea) for DNA sequencing. The DNA sequence results were then compared with the reference gene sequences available in the NCBI database using BioEdit software (Hall 1999). The specific sequence IDs referenced include NC_000007.14 for *BRAF*, NG_007524.2 for *KRAS*, and NG_012113.2 for *PIK3CA*. The electropherogram was manually examined to identify mutations in *BRAF* exon 15 at codon 600, *KRAS* exon 2 codons 12 and 13, and *PIK3CA* exon 9 codons 542–549.

### 2.4 Immunohistochemical (IHC) Staining

Three‐micron FFPE tissue sections of AC cases were prepared on coated glass slides. Positive controls and dilutions of each primary antibody are shown in Table 2.

**TABLE 2:** Monoclonal primary antibodies and positive control.

Antibodies	Brands	Dilution (μL)	Positive control
BRAF V600E (VE1)	Ventana	1:100	Known melanoma case with *BRAF* V600E
p‐ERK1/2	#4376; Cell Signaling Technology	1:100	Oral squamous cell carcinoma
p‐mTOR	#2976; Cell Signaling Technology	1:100	Breast cancer: Invasive ductal carcinoma (IDC)

For BRAF V600E, IHC staining was performed using a fully automated assay with the Ventana BRAF V600E (VE1) mouse monoclonal primary antibody on the Ventana BenchMark ULTRA IHC/ISH automated slide stainer, combined with the Ventana OptiView DAB IHC Detection Kit (Ventana‐Roche Diagnostics, Meylan, France). The U Optiview DAB IHC v6 CDx (v1.00.0135) procedure was performed according to the manufacturer's protocol.

Regarding p‐ERK1/2 and p‐mTOR immunostaining, samples were heated in 1× Target Retrieval Solution at pH 6.8 (S1699; Dako, Santa Clara, USA) for 13 min to retrieve antigen epitopes, before blocking endogenous peroxidase with 3% H_2_O_2_ for 10 min. Primary antibodies were diluted with Diluent solution (S0809; Dako, Santa Clara, USA) according to the dilutions listed in Table 2. The sample slides were incubated with primary antibody in a humidified chamber at 4°C overnight. Subsequently, they were incubated with Envision (K5007; Dako, 2600 Glostrup, Denmark) for 30 min at room temperature in a humidified chamber. The color reaction was generated using the Liquid DAB Plus Substrate‐Chromogen System (K5007; Dako, 2600 Glostrup, Denmark), followed by counterstaining with Mayer's hematoxylin.

### 2.5 Protein Expressions Analysis

Each IHC slide was captured at 400× magnification to randomly capture three to five tissue areas using a digital camera microscope. IHC staining images were evaluated independently by two investigators (J.P. and P.L.). BRAF V600E immunostaining was considered positive when cytoplasmic staining was present in more than 80% of tumor cells (Tun et al. 2025). On the other hand, p‐ERK1/2 and p‐mTOR were analyzed using a semi‐quantitative scoring method. Regarding the previous scoring method (Zheng et al. 2011): the intensity of stained cells was classified as negative (0), weak (1), moderate (2) and strong (3). The percentage of stained cells was determined as 0 (negative), 1 (positive less than 10%), 2 (positive approximately 10%–50%), 3 (positive approximately 51%–80%) and 4 (positive more than 80%). Immunoreactive Score (IRS) was calculated by multiplying the percentage positivity and intensity of stained cells. Notably, staining of the cytoplasm and/or nucleus in tumor cells of both proteins was considered positive. Interobservers correlation was assessed using the Kappa test (value = 0.814), indicating near‐perfect concordance.

### 2.6 Statistical Analysis

Descriptive statistics were used to analyze demographic data, the frequency of gene mutations, and associated protein expressions.

## 3 Results

Among 10 AC cases, the majority were males (7/10), with ages ranging from 25 to 60 years (mean age: 43.70 ± 13.17). The majority of tumors were found in the maxilla (7/10), with the plexiform type being more prevalent (6/10), as shown in Table 3.

**TABLE 3:** Demographic data and molecular results of ameloblastic carcinoma.

Demographic data					Molecular results					
No	Sex	Age (year)	Location	Histology sub‐type	*BRAF*	BRAF V600E	*KRAS*	p‐ERK1/2	*PIK3CA*	p‐mTOR
1	M	25	Mandible	Plexiform	**Mt**	+	Wt	^a^	Wt	^c^
2	M	60	Maxilla	Follicular	Wt	−	Wt	^b^	Wt	^b^
3	M	28	Maxilla	Follicular	**Mt**	+	**Mt**	−	**Mt**	^b^
4	F	57	Maxilla	Follicular	Wt	+	Wt	−	Wt	^a^
5	F	43	Maxilla	Plexiform	**Mt**	−	**Mt**	^a^	Wt	^b^
6	F	48	Maxilla	Plexiform	Wt	−	**Mt**	^b^	Wt	^c^
7	M	36	Maxilla	Plexiform	**Mt**	+	Wt	−	**Mt**	^b^
8	M	55	Mandible	Plexiform	Wt	−	Wt	−	**Mt**	^b^
9	M	55	Maxilla	Follicular	Wt	−	Wt	−	Wt	^b^
10	M	30	Mandible	Plexiform	Wt	−	Wt	−	Wt	^c^

*Note:* *BRAF*, *KRAS*, and *PIK3CA* were investigated by DNA sequencing. BRAF V600E, p‐ERK1/2, and p‐mTOR were analyzed by immunohistochemistry.

Abbreviations: −, negative; +, positive; F, female; M, male; **Mt**, mutation; Wt, wild‐type.

^a^ Mild.

^b^ Moderate.

^c^ Strong.

### 3.1 Identification of Oncogene Mutations in AC

All three oncogenic mutations were heterozygous, indicating the presence of both mutated and wild‐type alleles, as shown in Figure 2. The *BRAF V600E* missense mutation (Figure 2a) was found in 4/10 of cases, while 6/10 cases exhibited wild‐type *BRAF* (Table 3). The majority of AC cases (7/10) displayed wild‐type *KRAS* exon 2. Notably, *KRAS* mutations were observed in 3/10 AC cases (Table 3), specifically as missense mutations at codon 12 (G12V) of *KRAS* exon 2, as shown in Figure 2b. Although 7/10 AC cases exhibited wild‐type *PIK3CA* exon 9, mutations in exon 9 were identified at codons 545–547, including E545K, Q546Q, and E547K (Figure 2c), accounting for 3/10 AC cases (Table 3). Importantly, 3/4 *BRAF‐*mutated cases exhibited additional genetic alterations, including concurrent *KRAS* and *PIK3CA* mutations in case no. 3, *KRAS* mutation in case no. 5, and *PIK3CA* mutation in case no. 7 (Table 3).

**FIGURE 2:**
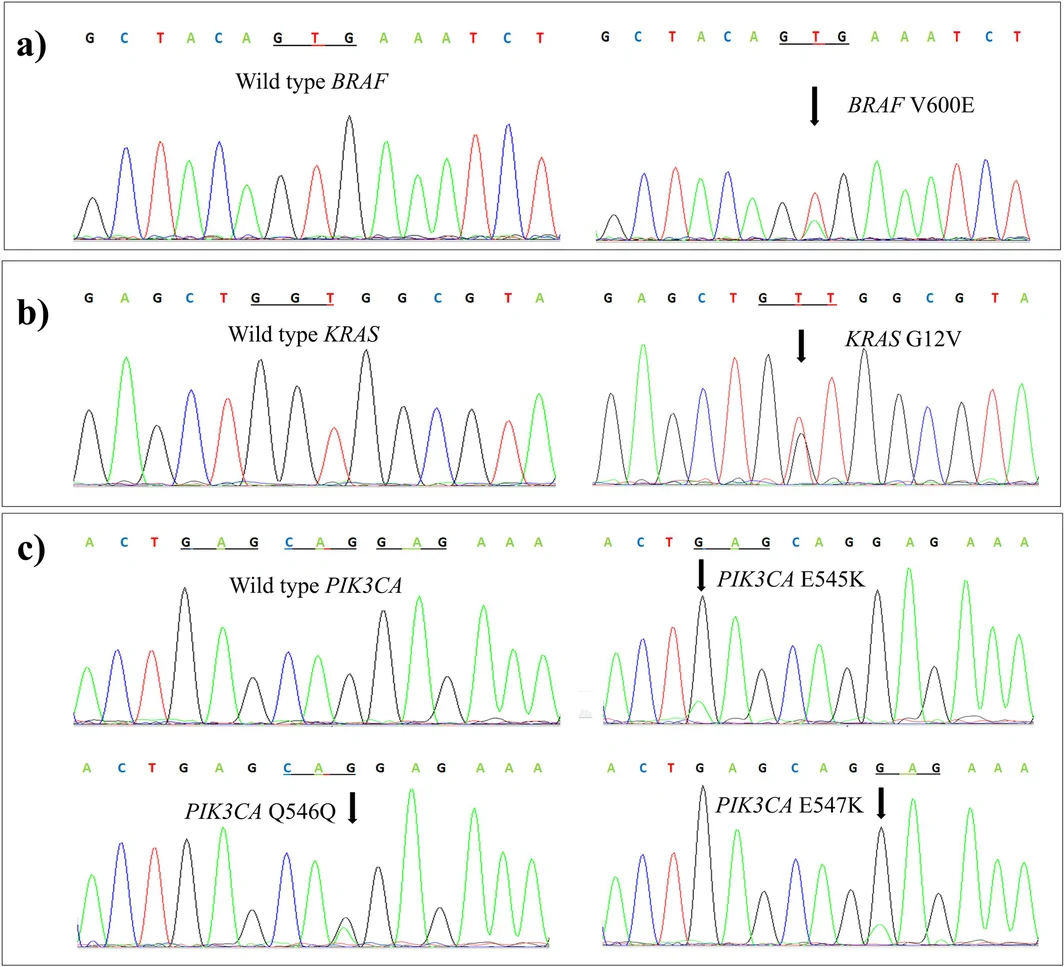
Gene mutations in ameloblastic carcinoma: (a) Electropherograms of wild‐type *BRAF* and *BRAF* exon 15 codon 600 mutation (V600E: GTG>GAG). (b) Electropherograms of wild‐type *KRAS* and *KRAS* exon 2 codon 12 mutation (G12V: GGT>GTT). (c) Electropherograms of wild‐type *PIK3CA* and *PIK3CA* exon 9 codons 545–547 (E545K: GAG>AAG, Q546Q: CAG>CAA, E547K: GAG>AAG). Arrows are pointing the heterozygous mutation in each gene.

### 3.2 Associated Protein Expressions in AC

Out of 10 AC cases, six cases (60.0%) were negative for BRAF V600E immunostaining. BRAF V600E was detected in four AC cases (40.0%), all of which showed strong immunostaining in tumor cells and some stromal cells, as shown in Figure 3a. For p‐ERK1/2 expression, four of 10 AC cases (40.0%) expressed this protein with mild to moderate immunostaining (mean IRS ± SD: 2.50 ± 2.53), as shown in Figure 3b. Regarding p‐mTOR expression, this protein was detected in all cases (10/10) at mild to strong immunostaining (mean IRS ± SD; 7.62 ± 2.87), which was observed in both the cytoplasm and nucleus of the ameloblastic tumor cells (Figure 3c), as well as in the stromal and endothelial cells. Notably, the two co‐mutation cases with concurrent *BRAF V600E* and *KRAS* mutations (case no. 3 and 5) demonstrated immunoexpression of only one of the two proteins (BRAF V600E or p‐ERK1/2), whereas the cases carrying both *BRAF V600E* and *PIK3CA* mutations expressed both associated proteins (Table 3).

**FIGURE 3:**
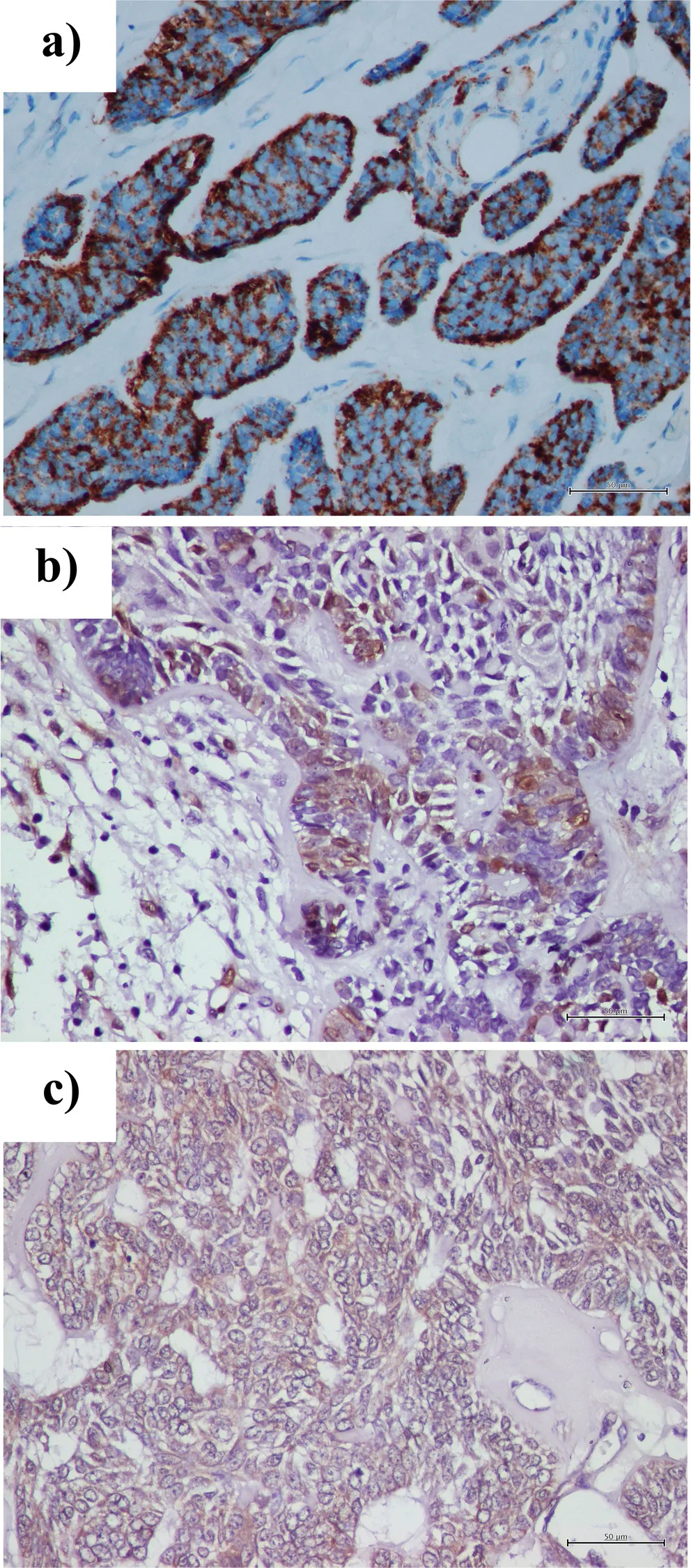
Protein expression in ameloblastic carcinoma (AC) by immunohistochemistry (400× magnification): (a) AC expressed strong immunostaining for BRAF V600E in cytoplasm of the tumor cells. (b) AC expressed mild immunostaining for p‐ERK1/2 in cytoplasm and nucleus of the tumor cells and stromal cells. (c) AC exhibited moderate immunostaining for p‐mTOR in cytoplasm of the tumor cells.

## 4 Discussion

In this study, the *BRAF V600E* mutation was detected in 40.0% of AC cases, with 40.0% also exhibiting BRAF V600E protein expression. However, one case demonstrated a discrepancy between the mutation analysis and protein expression results, indicating potential variability between the detection techniques. A previous study suggested that, while immunohistochemistry (BRAF V600E (VE1)) exhibits high specificity, its sensitivity is relatively low (Togni et al. 2022). Consequently, molecular testing is recommended to confirm the presence of *BRAF V600E* mutations in odontogenic tumors, ensuring a more accurate diagnosis (Togni et al. 2022). Given the biologically plausible evidence supporting a shared oncogenic pathway between AM and AC (Diniz et al. 2015; Oh et al. 2021), the presence of *BRAF V600E* mutations in a subset of AC cases may also suggest a possible origin from a pre‐existing AM. Nevertheless, because *BRAF* mutations are not exclusive to AM and can also occur *de novo* in AC (Niu et al. 2020), the presence of this mutation alone cannot conclusively determine tumor origin. Moreover, several reports have documented AC arising without a known pre‐existing AM and yet harboring *BRAF V600E* mutation (Diniz et al. 2015; Niu et al. 2020).

The *BRAF V600E* mutation has been widely recognized as a biomarker for AM and is commonly identified in previous studies (Buttner et al. 2023; Tun et al. 2025). This gene alteration highlights the potential of *BRAF V600E* as a therapeutic target for AM. Several case reports and small case series have documented clinical responses to BRAF inhibitors such as vemurafenib and dabrafenib in patients with recurrent or metastatic AM harboring *BRAF V600E* mutations (Brunet et al. 2019; Fernandes et al. 2018; Grynberg et al. 2024). Therefore, it is recommended that cases of AC with *BRAF V600E* mutations be considered for targeted drug therapy to improve treatment outcomes (Diniz et al. 2015). Consistent with our findings, previous studies have indicated that approximately 37%–40% of AC cases exhibit *BRAF V600E* mutations (Diniz et al. 2015; Oh et al. 2021). Additionally, the *BRAF V600E* mutation represents a prevalent genetic alteration that activates the MAPK pathway, which is essential for the proliferation, growth, and survival of cancer cells (Burotto et al. 2014). In our investigation, we noted diffuse cytoplasmic staining of BRAF V600E (VE1) in AC, highlighting the potential for selective use of MAPK‐targeted therapies. Collectively, these results indicate that the development of AC may be linked to the activation of the MAPK/ERK signaling pathway.

The *KRAS* exon 2 codon 12 mutation (G12V) was identified in 30.0% of AC cases, accompanied by mild immunostaining for p‐ERK1/2 (mean IRS ± SD: 2.50 ± 2.53). These findings suggest that KRAS‐mediated activation of MAPK/ERK signaling pathway may contribute partially to the pathogenesis of AC. However, the literature on AC pathogenesis involving this pathway remains limited, particularly regarding p‐ERK1/2 expression. To date, only two studies have examined proteins related to the MAPK/ERK signaling pathway, including Raf1, MEK1, ERK1/2, p38 MAPK, and ERK5 in AC cases (Kumamoto and Ooya 2007; Kumamoto et al. 2004). A previous study by Togni et al. (2022), which investigated gene mutations in 48 AM cases and 33 various odontogenic tumors, including five cases of AC, reported *KRAS* mutations in approximately 40.0% (2/5) of AC cases. Although these results align with our findings, they did not suggest a role for *KRAS* mutations in AC tumorigenesis (Togni et al. 2022). In contrast, *KRAS* mutations, specifically G12V and G12R variants, are commonly observed in adenomatoid odontogenic tumors, accounting for approximately 70.0% of cases (Coura et al. 2019). These mutations are associated with the activation of the MAPK/ERK signaling pathway, as evidenced by the expression of p‐ERK1/2, BRAF, CRAF, and KRAS proteins (Bologna‐Molina et al. 2019; Coura et al. 2019). Consequently, it has been suggested that *KRAS* mutations may drive the activation of the MAPK/ERK pathway, contributing to the tumorigenesis of adenomatoid odontogenic tumors, despite the limited number of studies available on this topic (Bologna‐Molina et al. 2019; Coura et al. 2019).

This study found that approximately 30.0% of AC cases exhibited *PIK3CA* exon 9 mutations, including two missense mutations (E545K and E547K) and one silent mutation (Q546Q). However, all cases exhibited a moderate level of p‐mTOR expression in tumor cells (mean IRS ± SD; 7.62 ± 2.87). The discrepancy between genetic mutations and their associated protein expression aligns with previous reports (Rahman et al. 2012) indicating that mTOR pathway activation does not always correlate with hotspot *PIK3CA* mutations. Several mechanisms may account for this discordance, including *PIK3CA* alterations in other exons (such as exon 20), loss or inactivation of *PTEN*, upstream activation of receptor tyrosine kinases or AKT, or compensatory signaling through MAPK–mTOR cross‐talk. These alternative drivers underscore that mTOR activation in AC may arise through multiple genetic or epigenetic mechanisms and therefore should not be inferred solely from exon‐specific *PIK3CA* mutation status.

By comparison, in head and neck tumors, approximately 3.70% (4/108) of OSCC cases exhibited *PIK3CA* exon 9 mutations at codons 542, 545, and 546, as well as mutations in exon 20. Additionally, a significant correlation between *PIK3CA* mutations and OSCC stage was observed, suggesting that these mutations occur later in OSCC development and play a critical role in cancer progression through the PI3K/mTOR signaling pathway (Kozaki et al. 2006). In AM, *PIK3CA* mutations have been reported in approximately 7.02% of cases (Gultekin et al. 2018). As for p‐mTOR expression, studies showed that 83.3% of AM cases expressed mTOR protein, which was associated with tumor aggressiveness. These findings suggest that mTOR may serve as a potential therapeutic target for AM (Li et al. 2023, 2012). In contrast to AM and OSCC, the study of the PI3K/mTOR signaling pathway in AC remains limited, highlighting the importance of these findings for advancing our understanding of AC pathogenesis.

Interestingly, three out of four *BRAF*‐mutated cases (75.0%) co‐harbored *KRAS* and/or *PIK3CA* mutations, and these cases also showed different expression of each associated protein. The presence of concurrent *BRAF*, *KRAS*, and *PIK3CA* mutations suggests that multiple oncogenic pathways are activated in these cases and may reflect pathway redundancy or compensatory activation. Typically, mutations in *BRAF*, *KRAS*, and *PIK3CA* occur independently, as reported in previous studies (Gultekin et al. 2018; Oh et al. 2022). However, this does not preclude the possibility of co‐mutations in our cohort. In a previous study, eight of 28 AM cases harbored *SMO* mutations together with *KRAS*, *BRAF*, or *FGFR2* mutations, as identified by paired‐end transcriptome sequencing (RNA‐seq) (Sweeney et al. 2014). Notably, one case in their series demonstrated a triple mutation involving *SMO*, *BRAF*, and *FGFR2*. Another study by Gultekin et al. (2018) reported that two of 38 *BRAF V600E*–mutated AM cases also harbored concurrent *PIK3CA* mutations. Moreover, co‐mutations of *BRAF* and *PIK3CA* are very rare; similar occurrences have been documented in other tumor types including colorectal cancer and thyroid carcinoma (Zou et al. 2014; Kang et al. 2025). These findings support the notion that while co‐mutations in tumors are uncommon, they are biologically plausible and may contribute to tumor aggressiveness and poorer clinical outcomes. Functional studies are warranted to validate the biological impact of gene and protein activation in AC cell models.

It is notable that the two cases with concurrent *BRAF V600E* and *KRAS* mutations (cases no. 3 and 5) show immunoexpression of only one of the two proteins, either BRAF V600E or p‐ERK1/2. This pattern raises the possibility that absent ERK1/2 phosphorylation reflects preferential activation of alternative pathways by *KRAS*, such as PI3K/AKT particularly in case no. 3, which also harbors a *PIK3CA* mutation and exhibits positive p‐mTOR staining. This finding suggests pathway bias or compensatory signaling within the tumor. Alternatively, low p‐ERK1/2 levels despite *KRAS* mutation may reflect spatial heterogeneity, phospho‐epitope degradation from fixation or processing, or sampling of regions with lower MAPK pathway activity.

The present study provides molecular insights into AC, a rare and understudied malignancy, by integrating both genetic mutation analysis and protein expression. The combined evaluation of *BRAF*, *KRAS*, and *PIK3CA* mutations alongside downstream pathway activation of MAPK/ERK and PI3K/mTOR offers a more comprehensive understanding of the tumor's molecular landscape. However, several limitations should be acknowledged. First, the sample size was relatively small (*n* = 10), reflecting the rarity of AC, and this may limit the generalizability of the findings. Second, the mutation analysis was restricted to selected genes, and other potentially relevant genetic alterations may have been missed. Third, the retrospective nature of the study and the use of FFPE tissues may have influenced DNA quality and mutation analyses. Further studies with larger cohorts and broader genomic approaches are warranted to validate and expand upon these findings.

In conclusion, our study suggests that the pathogenesis of AC involves the activation of both the MAPK/ERK and PI3K/mTOR signaling pathways. This is supported by the presence of *BRAF*, *KRAS*, and *PIK3CA* mutations, as well as the expression of BRAF V600E, p‐ERK1/2, and p‐mTOR in AC cases. Increasing the sample size in future studies is recommended to validate these findings, given the limitations of the small sample size and the low frequency of detected mutations. Additionally, it is advisable to conduct a more comprehensive examination of the genes and proteins associated with the PI3K/mTOR signaling pathway to further clarify and expand upon the findings presented in this study.

## Author Contributions

**Puangwan Lapthanasupkul:** conceptualization, investigation, validation and writing – review and editing. **Jintana Pankam:** methodology, data curation, investigation, formal analysis and writing – original draft. **Siribang‐on Piboonniyom Khovidhunkit:** funding acquisition, resources, supervision, and writing – review and editing. **Prapapun Seneetuntigul:** investigation. **Nakarin Kitkumthorn:** conceptualization, validation, supervision, project administration and writing – review and editing.

## Funding

The authors have nothing to report.

## Ethics Statement

This study was approved by the Faculty of Dentistry and Faculty of Pharmacy, Mahidol University, Institutional Review Board (COA no. MU‐DT/PY‐IRB 2023/071.1411).

## Consent

The authors have nothing to report.

## Conflicts of Interest

The authors declare no conflicts of interest.

## Data Availability

Data are available through the corresponding author upon request.
